# Comparison of a Label-Free Quantitative Proteomic Method Based on Peptide Ion Current Area to the Isotope Coded Affinity Tag Method

**DOI:** 10.4137/cin.s385

**Published:** 2008-04-17

**Authors:** Soyoung Ryu, Byron Gallis, Young Ah Goo, Scott A. Shaffer, Dragan Radulovic, David R. Goodlett

**Affiliations:** 1 Department of Medicinal Chemistry, University of Washington, Seattle, WA, U.S.A; 2 Department of Statistics, University of Washington, Seattle, WA, U.S.A; 3 Department of Mathematics, Florida Atlantic University, Boca Raton, FL, U.S.A

**Keywords:** label-free quantification, peptide ion current area (PICA), isotope coded affinity tag (ICAT), spectral count

## Abstract

Recently, several research groups have published methods for the determination of proteomic expression profiling by mass spectrometry without the use of exogenously added stable isotopes or stable isotope dilution theory. These so-called label-free, methods have the advantage of allowing data on each sample to be acquired independently from all other samples to which they can later be compared *in silico* for the purpose of measuring changes in protein expression between various biological states. We developed label free software based on direct measurement of peptide ion current area (PICA) and compared it to two other methods, a simpler label free method known as spectral counting and the isotope coded affinity tag (ICAT) method. Data analysis by these methods of a standard mixture containing proteins of known, but varying, concentrations showed that they performed similarly with a mean squared error of 0.09. Additionally, complex bacterial protein mixtures spiked with known concentrations of standard proteins were analyzed using the PICA label-free method. These results indicated that the PICA method detected all levels of standard spiked proteins at the 90% confidence level in this complex biological sample. This finding confirms that label-free methods, based on direct measurement of the area under a single ion current trace, performed as well as the standard ICAT method. Given the fact that the label-free methods provide ease in experimental design well beyond pair-wise comparison, label-free methods such as our PICA method are well suited for proteomic expression profiling of large numbers of samples as is needed in clinical analysis.

## Introduction

Genetic or environmental perturbations of an organism can lead to changes in protein expression. The traditional stable isotope dilution theory (De Leenheer, 1992) was first utilized in the proteomic field in 1998 to determine quantitative changes in protein expression by mass spectrometry (Chait et al. 1980; Gygi et al. 1999). Since then, many variations on the stable isotope-labeling theme have been developed to measure relative protein abundance in complex samples (Patterson and Aebersold, 2003). Quantitative proteomic profiling using isotope-coded affinity tags (ICAT) (Gygi et al. 1999) and the many variants developed since are widely used to reveal altered protein expression levels between two different samples (Patterson and Aebersold, 2003). Subsequently, automated statistical algorithms, such as ASAPRatio (Li et al. 2003) and RelEx (MacCoss et al. 2003), have also been developed to interpret data and enhance analysis of these complex data sets.

Recently, several labeling methods were developed to overcome the disadvantage of labeling methods like ICAT. One notable disadvantage of ICAT is that it fails to quantify proteins with no cysteine residues. Several publications have suggested alternative chemical modification strategies to overcome these problems: e.g. the amino-reactive labeling strategy ICPL (isotope-coded protein label; (Schmidt et al. 2005)) and the successor to ICAT, iTRAQ (Hardt et al. 2005). Another disadvantage of methods like ICAT is that only two samples may be compared for a given analysis. To overcome such limitations, an improved approach called iTRAQ was developed (Hardt et al. 2005) which allows four or eight samples to be profiled in one experiment. Unlike the limitation of ICAT for cysteine-only containing peptides, the iTRAQ chemistry can be applied to samples with a high degree of complexity independent of their amino acid compositions (Hardt et al. 2005). However, the iTRAQ method is still limited by the number of samples that can be compared in a single experiment. In addition, the combination of many samples into one for mass spectrometric analysis will logically reduce the measurable dynamic range because each mass spectrometry experiment used to read out the proteins and their abundance levels in the sample is limited to a given amount of sample that may be analyzed. Further, because the marker ions (i.e. readout) utilized in iTRAQ method reside at low *m/z* values, the method is not well suited for analysis on ion trap instrumentation, a common platform for proteomic analysis. While the popular in vivo labeling strategy, SILAC (Ong et al. 2002), circumvents some of the initial problems with ICAT, it too suffers from reagent cost and difficulty in conducting higher-order comparisons. Primarily for these reasons, researchers became interested in label-free methods which made experimental design well beyond pair-wise comparisons possible. Additionally, label-free approaches also require no reagents and hence greatly simplify sample preparation and reduce experimental cost.

To date several papers have reported the use of label-free quantification to profile protein expression in complex protein mixtures. These methods consist of two basic types which use either MS1 precursor ion (i.e. MS survey scan) data or MS2 tandem mass spectrometry data (i.e. MS/MS) to estimate changes in relative abundance or proteins between samples. The MS1 based methods associate changes in relative protein abundance from direct measurement of peptide ion current areas (Radulovic et al. 2004; Silva et al. 2005; Wang et al. 2003; Wiener et al. 2004; Guina et al. 2007). The MS2 based methods estimate differences in relative protein expression by either accounting for the extent of protein sequence coverage or the number of tandem mass spectra generated, also known as spectral counting (Colinge et al. 2005; Liu et al. 2004; Tang et al. 2006; Old et al. 2005; Zybailov et al. 2006; Cox et al. 2007). All these studies demonstrate the feasibility of label-free methods to reflect relative changes in protein abundance between samples. However, most of these studies lacked rigorous validation, error calculation, and report of false positive rates. To confirm that results from label-free methods were as accurate as stable isotope based methods, we conducted a comparative analysis between the ICAT method and our in-house developed label-free method.

In this study, we report the design and use of peptide ion current area (PICA) software for label-free quantification. The algorithm is automated and based on peptide-specific extraction of MS1 data to calculate changes in relative protein abundance between samples of interest. Specifically, the PICA algorithm calculates area under the curve generated by plotting a single ion current trace for each peptide of interest and compiles measurements for individual peptides into corresponding protein values. We examined the performance of the PICA algorithm by three different experimental approaches: 1) analysis of standard protein mixtures containing known concentrations of protein, 2) comparison to ICAT results to determine the relative known concentrations of standard proteins, and; 3) analysis of bacterial lysates containing known concentrations of three standard proteins. In addition, we compared the MS1 based PICA method to the MS2 based spectral counting method (Colinge et al. 2005; Liu et al. 2004) accuracy in quantification.

## Materials and Methods

### Preparation of seven-protein standard mixtures

Seven individual protein stock solutions were prepared at 100 μM in phosphate buffered saline (PBS). The proteins used were purchased from Sigma (St. Louis, MO) and included bovine catalase (BC), chick ovalbumin (CO), bovine β-lactoglobulin (BL), horse myoglobin (HM), Aspergillus oryzae α-amylase (AA), bovine apotransferrin (BAT), and bovine serum albumin (BSA). These stock solutions were mixed to form three different protein samples each containing all of these proteins but where some vary in concentrations according to the following scheme where X = 100 μM: Sample 1 = 5 proteins equimolar + BAT at 0.25X + BSA at 1X; Sample 2 = 5 proteins equimolar + BAT at 0.5X + BSA at 0.5X; and Sample 3 = 5 proteins equimolar + BAT at 1X + BSA at 0.25X. We refer to these samples later on in the text as Samples 1, 2, and 3. These three samples were used for both ICAT and label free analyses.

### Proteolysis of protein standards

Protein mixtures were denatured by titrating in urea until the concentration reached 6M and the pH was adjusted to 8.3. They were then reduced by bringing the solution to 5 mM TCEP (Tris(2-carboxyethyl)phosphine HCl) and incubated for 1 hr at 37 ºC after which proteins were alkylated by addition of iodoacetamide to a concentration of 20 mM and again incubated for 1 hr at RT in the dark. The reaction was quenched by addition of dithiothreitol to 20 mM and incubated for 1 hr at RT. The protein mixture was then diluted 10-fold in 25 mM ammonium bicarbonate and digested with Promega (Madison, WI) sequencing grade trypsin at an enzyme:substrate ratio of 1:100 (w/w) followed by incubation overnight at RT. The mixture of peptides was taken to dryness in a speedvac, dissolved in 5% acetonitrile/0.1% trifluroacetic acid, and de-salted on a Macrospin Vydac silica C18 column (The Nest Group, Southborough, MA). Each sample was analyzed six times by liquid chromatography mass spectrometry (LC-MS).

### Preparation of an isotope-coded affinity tag (ICAT) labeled peptides

Aliquots of 500 μg total protein were labeled as described using a Cleavable ICAT^TM^ Reagent Kit (Applied Biosystems, Foster City, CA). Briefly, each of the three seven-protein standard mixtures described above were denatured and reduced in 0.05% SDS, 5 mM EDTA, 200 mM Tris-HCl (pH 8.3), 6 M urea, and 5 mM TCEP-HCl for 30 min at RT. Isotopically heavy or light ICAT reagent was added to the reduced protein solution at a final concentration of 1.75 mM and incubated for 2 hrs at 37 °C. The ICAT labeling reaction was quenched by DTT at a concentration of 12 mM for 5 min at RT. Heavy or light isotope labeled samples were combined, and the urea concentration was reduced to 1 M by dilution with water. Trypsin (sequencing grade modified, Promega, Madison, WI) was added at a ratio of enzyme:substrate of 1:50 (w/w) and the sample was incubated overnight at 37 °C. The peptide mixture was diluted with an equal volume of Buffer A (5 mM KH_2_PO_4_, 25% CH_3_CN), adjusted to pH 3 with 10% H_3_PO_4_, and desalted on a strong cation-exchange cartridge supplied with the cleavable ICAT kit (Applied Biosystems). Peptides labeled with the ICAT reagent were purified from non-labeled peptides using a monomeric avidin cartridge (Applied Biosystems). The ICAT-labeled peptides were eluted with 0.4% trifluoroacetic acid in 30% acetonitrile, and taken to dryness in a speed-vac. Finally, the biotin tag was cleaved from the peptides in 95% reagent A and 5% reagent B (both supplied as part of proprietary ICAT kit from Applied Biosystems) prior to mass spectrometric analysis. Normal protocol would be to further purify the ICAT-labeled peptides by strong cation exchange. In order to simplify the LCMS comparison this was not done and the ICAT-labeled peptides were analyzed as a single mixture three times by LC-MS/MS.

### Preparation of spiked whole cell lysate

*Francisella tularensis subspecies novicida* (Ellis et al. 2002) was grown in culture to mid-log phase at either 21 °C or 37 °C to mimic growth in insects and mammals, respectively. Cultures were centrifuged, the pellet containing bacteria washed twice in ice-cold PBS, and sonicated in PBS 5X (10 sec). Cell lysates were centrifuged at 5,000X g for 10 min at 4 ºC and the supernatant used as a whole cell lysate. Chick ovalbumin (CO), bovine catalase (BC), and bovine serum albumin (BSA) were added (i.e. spiked) to the whole cell lysate in varying ratios to create six unique samples. Each of the six samples had the same amount of *Francisella novicida* protein, but varying amounts of the three standard proteins. Furthermore, the three standard proteins were spiked into the whole cell lysates from growth at either 21 ºC or 37 ºC. For the whole cell lysates from bacteria grown at 21 ºC, three samples were prepared using X = 0.24 μM where 1a) CO 1.05X + BC 1.05X + BSA 4.20X, 1b) CO 1.05X + BC 3.15X + BSA 2.10X, and 1c) CO 1.00X + BC 6.00X + BSA 1X. For the whole cell lysates from bacteria grown at 37 ºC, three samples were prepared where: 2a) CO 1.00X + BC 1.00X + BSA 6.00X, 2b) CO 1.05X + BC 2.10X + BSA 3.15X, and 2c) CO 1.05X + BC 4.20X + BSA 1.05X. To avoid addition of too much of the standard proteins relative to the whole cell lysate, protein range-finding experiments were performed after which the standard proteins were spiked in to the lysate at a level ≤ the most intense *F. novicida* peptide ion current intensities. The sample was then prepared for LC-MS analysis as described above for the protein standard mixtures. All six of the samples, 1a-c and 2 a-c, were analyzed by LC-MS/ MS in triplicate.

### Reversed-phase HPLC and tandem mass spectrometry

Here LC-MS refers to a generic reversed phase HPLC-tandem mass spectrometry experiment conducted on a hybrid linear ion trap-Fourier transform-ion cyclotron resonance (LIT-FT-ICR) mass spectrometer (LTQ-FT; Thermo Electron Corp., San Jose, CA) where precursor ion scans (MS1) occur in the FT-ICR simultaneous and in parallel to tandem MS scans (MS2) in the linear ion trap (LIT). For clarity we designate data derived from precursor ion scans as MS1 and from peptide tandem MS scans as MS2. All peptide mixtures were analyzed by HPLC-electrospray ionization (ESI) MS operating in the positive ion mode. Nanoflow HPLC was performed using a Paradigm MS4B MDLC (Michrom Bioresources, Auburn, CA) coupled to a Paradigm Endurance autosampler (Michrom Bioresources). A homemade precolumn was employed consisting of a 100 μm i.d. fused silica capillary packed with ~2 cm of 200 Ǻ (5 μm C18 particles (C18AQ; Michrom) and held in place by a sintered glass frit (Lichrosorb 60 Ǻ (5 μm) Si; Varian, Palo Alto, CA). Peptides were separated on a home-made analytical column consisting of a 75 μm i.d. fused silica capillary with a gravity-pulled tapered tip packed with ~11 cm of 100 Ǻ (5 μm C18 particles (C18AQ; Michrom)). With an injection volume of 10 μL, peptides were loaded on the precolumn at ~10 μL/min in water/acetonitrile (95/05) with 0.1% (v/v) formic acid. Peptides were eluted using an acetonitrile gradient flowing at ~220 nL/min using mobile phase consisting of H_2_O, acetonitrile, both containing 1% (v/v) formic acid. The ESI voltage was applied via a liquid junction using a gold wire inserted into a micro-tee union (Upchurch Scientific, Oak Harbor, WA) located between the pre- and analytical columns. Precursor ion *m/z* was measured in the FT-ICR where resolution was set to 100,000 (*m/z* 400) and ion populations held at 10^6^ via automatic gain control (AGC). Tandem mass spectra were acquired in the linear ion trap where ion population was set to 10^4^. Data were acquired using an MS1 or “survey” scan in the FT-ICR followed by data-dependent tandem MS (MS2) in the linear ion trap of the three most abundant precursor ions from the prior MS1 scan. For the standard protein digests, an exclusion mass list was used to limit MS2 data redundancy. For the spiked whole cell lysate, singularly-charged ions were excluded from data-dependent ion selection. In either case data redundancy was minimized further by excluding previously selected precursor ions (−0.1 Da/ + 1.1 Da) for 60–120 seconds following their selection for MS2. Data were acquired using Xcalibur, version 1.4 (Thermo Electron Corp.).

### Peptide and protein identification

All tandem mass spectra acquired in the LTQ were subjected to database search using SEQUEST (Eng. J. K.; McCormack, 1994). Confidence in matches was generated using Peptide-Prophet scores (Keller et al. 2002) of correct identification probability > 0.8 as well as Protein-Prophet scores (Nesvizhskii et al. 2003) of correct identification probability > 0.8. Peptide sequences with a contribution of peptide i to corresponding protein n, *w_i_^n^* < 0.99 (Nesvizhskii et al. 2003) were culled from the data set because peptide sequences corresponding to multiple proteins were not appropriate for protein expression estimation. In other words, the peptide sequences that map to more than one protein accession due to protein sequence homology were not used for quantification. Only tandem mass spectra that matched to peptide sequences with the above stated confidence were used for the further analysis.

## Data Normalization

Quality and reproducibility of LC-MS1 data were monitored by overlaying ion maps (*m/z* vs. retention time) for multiple data sets using The Dragon™ visualization tool (Radulovic et al. 2004). The data were then normalized by two different approaches. The first approach employed *m/z* peak ion intensity (Foss et al. 2007). The data normalization was processed as follows: for each *m/z* region (grouped in 10 m/z increments, e.g. 400–410 m/z), the intensity value *I* was calculated by combining the median and trimmed mean (average after discarding the top 2%) of all ion intensities in the region. Then, the intensity value *I* was smoothed by fitting the robust linear regression (*I* = *a* + *b* · *m*/*z*). Then, the individual m/z ion intensity values were normalized by using *Î*.

Secondly, the data were normalized via chromatographic retention time. The following information was gathered for each identified peptide: i) peptide sequence, ii) corresponding parent protein, iii) peptide molecular weight, iv) theoretical *m/z* (for the first 3 isotopes), v) charge-state, and vi) elution time(s). All peptides in the beginning (< 10 minutes) and end (> 65 minutes) of the chromatographic run were filtered out. In cases where a single unique peptide appeared to elute at multiple discrete retention times, only the major chromatographic peaks were used to estimate the peptide expression level. The chromatographic peak was considered as major, if it had the average intensity larger than 1,000 times the other chromatographic peaks with the same peptide identification by database search and charge state. Retention time was normalized between experiments using peptides with higher confidence (Peptide Prophet probability > 0.9) by employing the linear regression model (predictor variables: retention time and *m/z*, response variable: retention time shift). Once the retention time was normalized, individual peptide expression levels were obtained by measuring the area under the curve generated by plotting single ion current traces.

### Protein relative expression determined from peptide ion current area (PICA)

**Figure 1:**
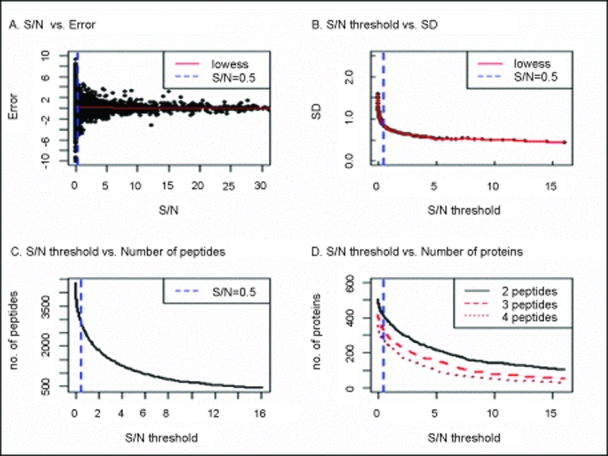
Signal to background noise (S/N) threshold Scatter-plots used to calculate signal to background noise (S/N) threshold comparisons to (**A**) the logarithm of peptide ratio error for each peptide; (**B**) their corresponding standard deviations (SD) from (A); (**C**) number of peptides; and (**D**) number of proteins.

**Figure 2:**
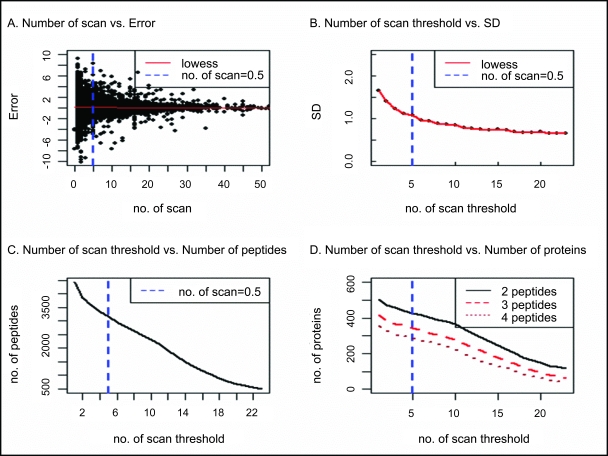
MS1 scan number threshold Scatter-plots used to calculate MS1 scan number thresholds comparisons to (**A**) the logarithm of peptide ratio error for each peptide; (**B**) their standard deviations (SD) from (A); (**C**) number of peptides (**D**) number of proteins.

Protein expression levels were determined by the summation of peptide chromatographic peak areas using in-house developed software described herein. The peptide ion current area (PICA) method utilizes measured areas under a curve determined from reconstructed ion chromatograms (RIC) of MS1 data for select peptides identified by tandem mass spectrometry. To draw these RIC curves the theoretical *m/z* of first three isotope peaks were used. Initially chromatographic peaks within + /− 1.5 minutes and + /− 0.0015 *m/z* were extracted along with the background area. The chromatographic peaks were smoothed by a Savitzky-Golay filter (Press, 1992) with a second-order polynomial smoothing and a moving window of width 7. In other words, the intensity at scan i was replaced by a new value which was obtained from polynomial fit (i.e. parabola) to 7 neighboring points. These 7 neighboring points were intensity values at scan i, at 3 scans before scan i, and at 3 scans after scan i. By this smoothing process, the level of noise was reduced without much biasing of intensity values. After this smoothing process, the scan h was determine as the starting point of scan where *h* = min(*S*), S = {i: *i* ≤ *d*, *d* = observed/predicted scan of peptide, intensity at scan *t* ≥ *x* for ∀*t* where *i* ≥ *t* ≥ *d*}. In our analysis, we use *x* = 0 and in a similar way, the last scan number of a peak was determined. Peptide expression level was then determined by measuring the chromatographic peak area of the corresponding peptide. The S/N is estimated by dividing the chromatography area of the peptide by the sum of all other peaks within the initial retention time (RT) window ( + /− 1.5 minutes) and m/z window defined above. This measure reflects not only noise but also co-eluting peptides. The plots shown in Figures 1 and 2 were used to determine the following filtering thresholds: 1) a minimum signal to noise ratio of 0.5 and 2) a minimum number of scans at which a peptide elutes was set at five for simple mixture and three for complex. The peptide expression levels that passed the previously specified thresholds were then summed (for all charge states of a given peptide and for all peptides for a given protein) to obtain individual protein abundance values which were subsequently log-transformed. A two sided two sample t-test with unequal variance was performed and multiple testing errors were corrected by using a false discovery rate (Benjamini and Hochberg, 1995). By this procedure, the protein ratios along with the adjusted p-values are obtained to help researcher to determine which proteins are differentially expressed.

### Protein relative expression determined by spectral counting and ICAT analysis

Tandem mass spectra that passed filtering criteria described in the previous section (peptide and protein identification) were counted for the purpose of comparing the spectral counting (Colinge et al. 2005; Liu et al. 2004) approach to our label-free method. Proteins with an average spectral count > 2 were included for quantification.

Quantitative information encoded in ICAT data was retrieved by analysis using ASAPRatio (Li et al. 2003) and XPRESS (Han et al. 2001). Briefly, XPRESS reconstructs the heavy- and light-isotope elution profiles of the MS1 precursor ions to estimate the relative abundance of proteins. ASAPRatio is similar to XPRESS, but statistical methods such as Savitzky-Golay smoothing filters (Press, 1992), and Dixon’s test for outliers are also employed (Dixon, 1953).

## Results and Discussion

### Compromise between accuracy and number of quantifiable proteins

Even after cautious sample preparation and mass spectrometry analysis, random (experimental) variation will be present in any data set. A standard way to reduce these errors is by applying high filtering thresholds, but this will decrease the number of proteins that are quantified. In order to determine exactly what thresholds to use for our validation experiments we examined how changes in minimum signal to noise (S/N) ratios and raw MS1 scan numbers (a function of chromatographic elution time) affected our results. From the plots shown in Figures 1 and 2 we determined that the following filtering thresholds of 0.5 for a minimum S/N as well as five and three scans utilized as the minimum raw MS1 scans for simple and complex mixtures, respectively, should be used. As can be seen in Figure 1A and 1B the peptide quantification levels with a higher S/N have smaller error than ones with a low S/N. This is intuitively correct because it is expected that data quality will degenerate as the signal becomes less distinguishable from the background noise. Additionally, we expect that a peptide that elutes over a longer time chromatographically, but not longer than the average peptide chromatographic peak width which may indicate chromatographic problems, will have smaller errors in quantification (Fig. 2A and 2B) than one present in very few scans which might indicate that there is not enough peptide present to accurately quantify. Each of these parameters, S/N and peptide chromatographic peak width, is a function of the amount of peptide in the sample and the ability of mass spectrometer to measure it. Furthermore, we note that when higher stringent threshold values are applied as shown in Figures 1C and 2C, then the number of peptides that can be quantified will decrease. Additionally, as Figures 1D and 2D show this also results in a decrease in the number of proteins that may be quantified. This compromise between errors in quantification and number of proteins that may be quantified, is a trade-off that the experimentalist must consider when analyzing their data. Logically, filtering thresholds must be determined in the context of a “fitness of purpose” for the study. In our examples we chose modest values (S/N threshold = 0.5 and a minimum of 5 MS1 scans for simple mixture) because we simply intended to validate our PICA method against the standard ICAT and a common spectral counting method.

### Comparison of ICAT and label-free methods on a seven-protein mixture

For a proof of concept test of our label-free PICA method versus the stable isotope based ICAT method, both of which use MS1 data, three unique seven-protein standard mixtures were prepared. These three mixtures were compared in a pair-wise fashion using both ICAT and PICA software. Specifically, PICA was used to compare Sample 1 vs. Sample 2, Sample 1 vs. Sample 3 and Sample 2 vs. Sample 3 whereas ICAT analysis was done on only two pairs: Sample 1 vs. Sample 2 and Sample 1 vs. Sample 3. We then compared how well each of the two methods measured the known protein concentrations. A protein ratio for the ICAT-labeled 7-protein mixture was obtained using both XPRESS (Han et al. 2001) and ASAPRatio (Li et al. 2003). Mean squared errors (MSE) were calculated for the XPRESS and ASAPRatio results and compared to MSE’s calculated from the spectral count and the PICA methods. Calculated MSE values represent error between the observed and actual ratios. In this case MSE values were calculated for each two-sample comparison by subtracting the log of the actual ratio of the protein from the log of observed ratio of the protein. Note that a lower MSE value is achieved when the observed ratio is closest to the actual ratio.

**Figure 3:**
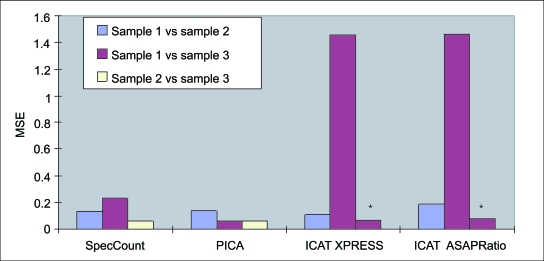
Comparison between label-free and ICAT methods The three different concentrations of the seven-protein mixtures were pair-wise compared and the differential expression gauged by four different quantification methods (spectral counting, PICA, XPRESS, and ASAPRatio). Bars with asterisks represent MSE for sample 1 vs. 3 after removing 2 proteins at 4 fold-differences.

Figure 3 displays the MSE’s obtained from the four different quantification methods: spectral counting, PICA, and ICAT using both XPRESS and ASAPRatio software. No protein ratio was derived from the ICAT-labeled horse myoglobin protein because it did not contain any cysteine residues. For sample 1 vs. 2, there were two proteins at 2-fold actual difference and four proteins at the same concentration. The low MSE scores for each of the four methods suggest both the label-free and ICAT methods performed well in detecting a 2-fold differences. For the comparison of sample 1 vs. 3, there were two proteins present at a 4-fold difference in concentration and four proteins at the same concentration. Here large errors were found when these two samples were compared using XPRESS and ASAPratio to analyze the ICAT data. Notably, we observed protein ratios by XPRESS that were 1.14 and 1.06 when the actual ratios were 0.25 and 4.00. The observed protein ratios by ASAPRatio were 0.95 and 0.88 when again the actual ratios were for 0.25 and 4.00. As shown by the bars with asterisks in Figure 3 both ICAT software methods performed well in detecting the four proteins at the same concentration. We cannot fully explain the poor performance by the ICAT software and method to detect the known 4-fold change, but it may be due to various reasons such as inefficient ICAT labeling and/or, unexpected suppression of ion current during the electrospray process. This result suggests label-free methods have outperformed the ICAT-labeled quantification for detecting 4-fold changes in this study. Finally, no ICAT data was generated for the comparison between samples 2 vs. 3 because it was not needed to validate the comparison to PICA.

### Label-free analysis of a seven protein mixture

Three different standard mixtures consisting of seven proteins were analyzed by two different label-free methods, our PICA method and a previously published spectral counting method. As shown in Table 1 and discussed below the accuracy of the measured difference in protein ratios determined by the two methods was very close. From the known concentrations of proteins in each standard mixture, actual ratios of proteins between samples were obtained and then compared to observed ratios. The MSE calculated from PICA results was 0.12, 0.014, and 0.07 for the comparison of sample 1 vs. 2, sample 1 vs. 3, and sample 2 vs. 3, respectively. The MSE calculated from the spectral counting method was very similar at 0.12, 0.20, and 0.04, for the same samples, respectively. Based on these calculated MSE values for the three comparisons, the PICA method performed slightly better than spectral counting. This is to be expected because the PICA method, based on direct measurement of area under a curve from MS1 data, should do a better job of accurately measuring the “amount” of a peptide present whereas the spectral counting approach is simply an estimate based on approximately randomly generated MS2 data (Liu et al. 2004; Colinge et al. 2005).

### Label-free analysis of *Francisella novicida* lysates spiked with standard proteins

**Figure 4:**
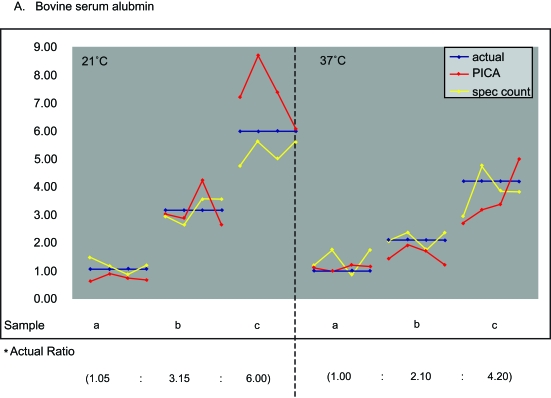

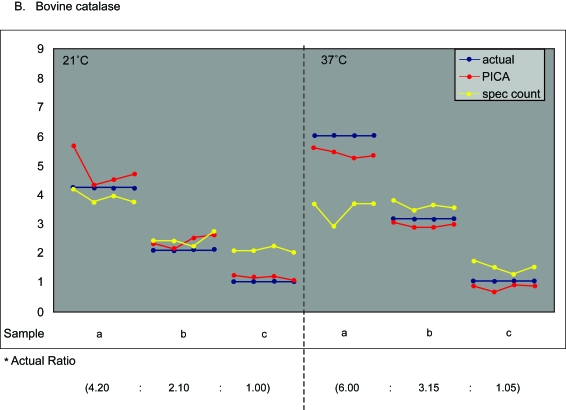

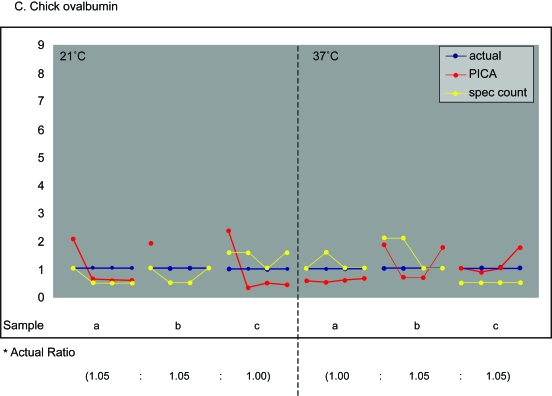
Comparison of label-free analysis of 3-protein standards spiked into a *Francisella novicida* lysate Bovine serum albumin (a), Bovine Catalase (b) and chicken ovalbumin (c) were spiked into a *Francisella novicida* lysate. Each data point represents either the actual (blue) spiked in value for the standard proteins or a value calculated four times from single technical LCMS analysis using either PICA (red) or spectral counting (yellow). **Note:** *The expected “actual” ratio from the 3 samples, a, b, and c.

Finally, we chose to carry out a more realistic experiment on a complex sample. For this we used whole cell lysates of *Francisella novicida* into which three proteins were added at known concentrations. For reasons related to the complexity of ICAT sample preparation and a desire to keep the number of samples low, we compared how well a previously published spectral counting method performed against PICA rather than ICAT analysis. All samples were analyzed by LC-MS/MS and the ability of both the PICA and spectral counting methods to detect the known difference in abundance for the 3-protein standard (Fig. 4). The actual protein abundance is indicated in Figure 4 by blue lines and the observed protein abundance determined by PICA and spectral counting shown as red and yellow lines, respectively. The three data sets on the left half of each of Figure 4A, 4B and 4C represent data obtained from bacterial lysates grown at 21 ºC and on the right at 37 ºC. The MSE’s for the three spiked in standard proteins were 0.13 and 0.27 for the PICA and spectral counting, respectively. Thus, overall the PICA performed slightly better than spectral counting in detecting the “actual” differential expression for the spiked protein standards.

Next, the three samples from 21 ºC *F. novicida* were used to test how effectively the label-free methods could detect the differentially expressed proteins in pair-wise comparisons: i.e. a vs. b; a vs. c; b vs., c (see Method: Preparation of a spiked whole cell lystate). Among these three pair-wise comparisons it is expected that we should be able to observe differences in expression for two of the spiked in proteins, bovine catalase and bovine serum albumin, among the hundreds of Francisella proteins identified, but not for chick ovalbumin which was held constant. From PICA analysis, a total of 505 *Francisella* proteins were detected above the predetermined thresholds and quantified (data not shown). Among those 505 proteins, we expected to observe the two spiked proteins as differentially expressed in the three pair-wise comparisons. At a 95% confidence level (i.e. 5% false discovery rate) we detected five of the six comparisons as different, but at a 90% confidence level all six comparisons were detected as differentially expressed. When an identical analysis was done using the spectral counting method, a total of *Francisella* 345 proteins were detected above the threshold and quantified (data not shown). In this analysis at the 95% confidence level we detected four of the six changes and at 90% five making spectral counting slightly less capable than PICA in detecting known differences in protein expression. The false discovery rate of differentially expressed proteins, bovine catalase and bovine serum albumin, in pair-wise comparisons is shown in Table 2. Note that both the PICA and spectral count methods were able to detect almost all changes in protein abundance in FN sample at the lower false discovery rate (5–10%). This result suggests that these two methods may detect the differentially expressed proteins in complex biological samples at almost equal confidence. Future investigations will be directed to verify whether low abundance proteins can be detected and if proteins with different physicochemical characteristics may give different results.

### Protein sequence homology

In a real biological experiment, especially in mammalian systems, protein sequence homology can be problematic. Even after use of parsimony grouping algorithms, such as ProteinProphet, many peptides may be present in several confidently identified proteins; e.g. when the same peptide sequence maps to more than one protein accession in the final list of confidently identified proteins. In our analysis, we focus on quantifying the standard proteins which do not have this homology problem. To do so we used the peptides matched to only one protein by filtering by the relative weight *w_i_^n^* (calculated by ProteinProphet (Nesvizhskii et al. 2003)). In brief, the relative weight *w_i_^n^* is calculated as follows: For each peptide i and each protein n, $w_{i}^{n}=P_{n}/\sum_{s=1,\ldots N_{s}}P_{s}$ obtained where *N_s_* is the number of proteins that peptide i is mapped to, and *P_n_* is the probability of protein n. So, if one peptide is present in several confidently identified proteins, then this peptide will have small relative weight, *w_i_^n^* , and will be removed from consideration for quantitative analysis. For an organism where there is a significant protein sequence homology problem, we suggest use of lower relative weight *w_i_^n^* (< 0.99). Future studies will be directed to find the optimum relative weight *w_i_^n^* threshold and how to allow peptides with higher *w_i_^n^* contribute more in protein quantification.

## Conclusion

We developed MS1 based label free software referred to as PICA that quantifies the relative protein expression levels among samples using ion intensity from derived from the LC-MS chromatographic peak areas of peptides. In order to provide guidelines for researchers to understand the affects of various filtering thresholds, we showed how the number of proteins one can identify and quantify is a function of both MS1 S/N and chromatographic quality. We also note the obvious trade-off between number of proteins quantified and variation in peptide expression levels. Additionally, in order to investigate the performance of several proteomic quantification methods, the PICA method was compared to another label free method, spectral counting, and the standard ICAT method for ability to accurately quantify the known differences in protein concentrations between samples with a relatively small false positive rate. When tested on simple mixtures of known proteins and known concentrations all three methods performed adequately even though the PICA method yielded the smallest MSE values. For a complex bacterial lysate, the PICA approach performed better than the spectral counting method for ability to quantify a larger number of proteins and detect known differences in the levels of proteins. Considering that the PICA approach is well suited to compare a large number of complex samples simultaneously and requires relatively simple sample preparation, our PICA method is well suited for proteomic expression profiling of real biological samples.

## Supplementary Material

**Table S1.** The identified peptides from seven-protein standard mixtures.

## Figures and Tables

**Table 1:** Label-free analysis of relative protein levels in seven-protein mixtures.

Protein	Expected ratio	PICA		Spectrum count	
		Ratio	95% CI*	Ratio	95% CI
**Comparison between sample 1 and sample 2**					
Catalase	1.00	0.80	(0.54,1.17)	0.96	(0.77,1.18)
Lactoglobulin	1.00	0.81	(0.71,0.94)	0.89	(0.68,1.16)
Transferrin	2.00	1.63	(1.55,1.72)	1.56	(1.15,2.08)
Albumin	0.50	0.63	(0.52,0.79)	0.84	(0.62,1.12)
Ovalbumin	1.00	0.70	(0.46,1.04)	0.90	(0.61,1.36)
Myoglobin	1.00	1.10	(0.82,1.45)	1.16	(0.94,1.43)
Amylase	1.00	0.74	(0.48,1.09)	0.86	(0.64,1.15)
**Comparison between sample 1 and sample 3**					
Catalase	1.00	0.76	(0.52,1.10)	1.02	(0.82,1.25)
Lactoglobulin	1.00	1.02	(0.91,1.15)	0.99	(0.75,1.28)
Transferrin	4.00	4.39	(3.87,5.00)	2.80	(2.06,3.74)
Albumin	0.25	0.29	(0.27,0.32)	0.53	(0.39,0.70)
Ovalbumin	1.00	0.91	(0.59,1.33)	0.99	(0.70,1.36)
Myoglobin	1.00	1.13	(0.80,1.60)	1.04	(0.77,1.43)
Amylase	1.00	0.88	(0.57,1.29)	1.06	(0.80,1.39)
**Comparison between sample 2 and sample 3**					
Catalase	1.00	0.95	(0.72,1.25)	1.06	(0.95,1.19)
Lactoglobulin	1.00	1.26	(1.09,1.46)	1.11	(0.96,1.27)
Transferrin	2.00	2.69	(2.38,3.05)	1.80	(1.51,2.14)
Albumin	0.50	0.46	(0.37,0.57)	0.62	(0.52,0.76)
Ovalbumin	1.00	1.29	(0.97,1.70)	1.09	(0.75,1.55)
Myoglobin	1.00	1.03	(0.75,1.45)	0.90	(0.66,1.23)
Amylase	1.00	1.19	(1.03,1.36)	1.24	(0.96,1.58)

* CI = Confidence Interval.

**Table 2:** False discovery rate for detection of proteins spiked in *F. novicida* lysate.

Protein	Sample 1 vs. Sample 2	Sample 1 vs. Sample 3	Sample 2 vs. Sample 3
**PICA** (no. protein tested = 505)*			
**Bovine Catalase**	p = 0.10	p = 0.05	p = 0.01
**Bovine serum albumin**	p = 0.04	p = 0.05	p = 0.01
**Spectral Count** (no. protein tested = 345)*			
**Bovine Catalase**	p = 0.08	p = 0.01	p = 0.05
**Bovine serum albumin**	p = 0.02	p = 0.01	p = 0.24

* Total number of proteins identified and quantified by each method.
