# Supplementary material for: Comparison of a Label-Free Quantitative Proteomic Method Based on Peptide Ion Current Area to the Isotope Coded Affinity Tag Method
Source: Cancer Inform. 2008 Apr 17;6:243–55. doi: 10.4137/cin.s385 (PMC2623286; doi:10.4137/cin.s385)
Supplement: Supplementary file 1 [file cin-6-0243-s1.doc]

**Supplementary Table 1**. The identified peptides from seven-protein standard mixtures.

| Protein | peptide sequence | minimum XCorr | minimum DeltaCn | median PICA |
| --- | --- | --- | --- | --- |
| Albumin | AADDKEACFAVEGPK | 3.49 | 0.57 | 1283 |
| Albumin | ADICTLPDTEK | 1.71 | 0.19 | 81699 |
| Albumin | AEFVEVTK | 1.81 | 0.39 | 4817 |
| Albumin | AIPENLPPLTADFAEDKDVCK | 1.99 | 0.45 | 43446 |
| Albumin | AKEYEATLEECCAK | 3.11 | 0.44 | 1747 |
| Albumin | ALTPDETYVPK | 1.28 | 0.35 | 146271 |
| Albumin | CAADDKEACFAVEGPK | 2.16 | 0.45 | 2653 |
| Albumin | CCAADDKEACFAVEGPK | 3.19 | 0.59 | 990774 |
| Albumin | CCTESLVNR | 1.83 | 0.57 | 1282 |
| Albumin | CFSALTPDETYVPK | 2.52 | 0.58 | 534346 |
| Albumin | CTEDYLSLIL | 2.07 | 0.61 | 19225 |
| Albumin | CTEDYLSLILN | 2.01 | 0.43 | 54873 |
| Albumin | CTEDYLSLILNR | 1.53 | 0.35 | 243063 |
| Albumin | DAFLGSFLY | 1.03 | 0.54 | 24282 |
| Albumin | DAFLGSFLYEY | 1.45 | 0.34 | 2869 |
| Albumin | DAFLGSFLYEYSR | 1.64 | 0.33 | 135756 |
| Albumin | DAIPENLPPLTADFAEDK | 2.75 | 0.69 | 6210 |
| Albumin | DCCEKQEPERNEC | 2.85 | 0.42 | 76780 |
| Albumin | DDPHACYSTVF | 1.15 | 0.31 | 89028 |
| Albumin | DDPHACYSTVFDK | 1.69 | 0.12 | 616661 |
| Albumin | DICTLPDTEK | 1.63 | 0.30 | 70211 |
| Albumin | DLGEEHFK | 1.26 | 0.31 | 580 |
| Albumin | EACFAVEGPK | 1.29 | 0.28 | 32201 |
| Albumin | ECCDKPLLEK | 1.78 | 0.19 | 12275 |
| Albumin | ECCHGDLLECADD | 1.68 | 0.21 | 8391 |
| Albumin | ECCHGDLLECADDR | 1.50 | 0.36 | 515082 |
| Albumin | ECCHGDLLECADDRADLA | 1.81 | 0.45 | 66959 |
| Albumin | ECCHGDLLECADDRADLAK | 2.39 | 0.47 | 107642 |
| Albumin | ETMREKVLTSSAR | 1.83 | 0.40 | 18991 |
| Albumin | ETYGDMADCCEK | 1.58 | 0.50 | 7765 |
| Albumin | EYEATLEECCAK | 1.93 | 0.46 | 884713 |
| Albumin | EYEATLEECCAKDD | 2.39 | 0.51 | 2359 |
| Albumin | EYEATLEECCAKDDPHACY | 2.78 | 0.64 | 36222 |
| Albumin | FGDELCK | 1.67 | 0.44 | 1157 |
| Albumin | FHADICTLPDTEK | 1.33 | 0.39 | 85846 |
| Albumin | FKDLGEEHFK | 2.53 | 0.54 | 5877 |
| Albumin | FKDLGEEHFKG | 3.01 | 0.46 | 1803 |
| Albumin | FLGSFLYEYSR | 2.68 | 0.59 | 21327 |
| Albumin | FLYEYSR | 1.41 | 0.21 | 5637 |
| Albumin | FSALTPDETYVPK | 2.73 | 0.47 | 74370 |
| Albumin | FSQYLQQCPFDEHVK | 3.40 | 0.64 | 271062 |
| Albumin | FTFHADICTLPDTEK | 2.42 | 0.34 | 9002 |
| Albumin | FVAFVDK | 1.93 | 0.09 | 8625 |
| Albumin | FYAPELL | 1.48 | 0.42 | 17679 |
| Albumin | FYAPELLYYANK | 2.13 | 0.62 | 9233 |
| Albumin | HADICTLPDTEK | 1.42 | 0.53 | 1692 |
| Albumin | HLVDEPQNLIK | 2.14 | 0.46 | 2437758 |
| Albumin | HLVDEPQNLIKQ | 3.26 | 0.52 | 10946 |
| Albumin | HPYFYAPELL | 1.77 | 0.38 | 117034 |
| Albumin | HPYFYAPELLYY | 1.30 | 0.43 | 3991 |
| Albumin | HPYFYAPELLYYA | 1.55 | 0.48 | 8087 |
| Albumin | KCCAADDKEACFA | 1.76 | 0.56 | 1497 |
| Albumin | KEYEATLEECCAK | 2.75 | 0.39 | 1532 |
| Albumin | KHLVDEPQNLIK | 1.88 | 0.48 | 10979 |
| Albumin | KQTALVELL | 1.51 | 0.28 | 137910 |
| Albumin | KQTALVELLK | 2.15 | 0.53 | 186139 |
| Albumin | KVPQVSTPTLVE | 1.63 | 0.30 | 3821 |
| Albumin | KVPQVSTPTLVEV | 1.90 | 0.31 | 81915 |
| Albumin | KVPQVSTPTLVEVS | 1.56 | 0.28 | 174251 |
| Albumin | KVPQVSTPTLVEVSR | 2.38 | 0.28 | 2171099 |
| Albumin | KYNGVFQECCQAEDK | 3.69 | 0.33 | 39661 |
| Albumin | LFTFHAD | 1.39 | 0.38 | 7721 |
| Albumin | LFTFHADIC | 2.19 | 0.60 | 211008 |
| Albumin | LFTFHADICTLPDTEK | 2.22 | 0.46 | 1580684 |
| Albumin | LGEYGFQN | 1.34 | 0.41 | 27128 |
| Albumin | LGEYGFQNA | 2.03 | 0.50 | 123063 |
| Albumin | LGEYGFQNAL | 1.35 | 0.35 | 72499 |
| Albumin | LGEYGFQNALIV | 1.75 | 0.30 | 6131 |
| Albumin | LGEYGFQNALIVR | 1.08 | 0.42 | 522717 |
| Albumin | LKECCDKPLLEK | 2.32 | 0.38 | 1556 |
| Albumin | LKPDPNTLCDE | 1.54 | 0.41 | 3182 |
| Albumin | LKPDPNTLCDEFK | 2.17 | 0.38 | 999544 |
| Albumin | LKPDPNTLCDEFKADEK | 1.75 | 0.33 | 744058 |
| Albumin | LKPDPNTLCDEFKADEKK | 1.77 | 0.38 | 40001 |
| Albumin | LLECADDRADLAK | 1.56 | 0.42 | 2040 |
| Albumin | LSLILNR | 1.81 | 0.18 | 2828 |
| Albumin | LTADFAEDKDVCK | 1.81 | 0.41 | 15519 |
| Albumin | LTPDETYVPK | 1.17 | 0.42 | 154701 |
| Albumin | LVDEPQNLIK | 1.61 | 0.48 | 4556 |
| Albumin | LVNELTEFAK | 2.35 | 0.57 | 3038687 |
| Albumin | LVVSTQTALA | 1.32 | 0.28 | 171141 |
| Albumin | MPCTEDYLSLILN | 2.41 | 0.26 | 21735 |
| Albumin | MPCTEDYLSLILNR | 2.63 | 0.52 | 3195 |
| Albumin | NECFLSHKDDSPDLPK | 2.87 | 0.23 | 270081 |
| Albumin | NELTEFAK | 1.70 | 0.47 | 7120 |
| Albumin | NFVAFVDK | 1.43 | 0.48 | 17056 |
| Albumin | PCFSALTPDETYVPK | 3.17 | 0.52 | 14531 |
| Albumin | PENLPPLTADFAEDKDVCK | 5.57 | 0.72 | 7469 |
| Albumin | PHACYSTVFDK | 1.82 | 0.42 | 20339 |
| Albumin | PLTADFAEDKDVCK | 3.50 | 0.64 | 9080 |
| Albumin | PNTLCDEFKADEK | 2.65 | 0.43 | 71561 |
| Albumin | PNTLCDEFKADEKK | 3.27 | 0.61 | 1077 |
| Albumin | PPLTADFAEDKDVCK | 1.65 | 0.42 | 10799 |
| Albumin | PQVSTPTLVEVSR | 1.83 | 0.58 | 6117 |
| Albumin | PTLVEVSR | 2.40 | 0.79 | 5786 |
| Albumin | QEPERNECFLS | 1.61 | 0.46 | 225289 |
| Albumin | QTALVELLK | 1.11 | 0.47 | 619705 |
| Albumin | QVSTPTLVEVSR | 1.69 | 0.51 | 164066 |
| Albumin | QYLQQCPFDEHVK | 2.12 | 0.27 | 26298 |
| Albumin | RHPEYAVSVL | 1.39 | 0.43 | 21544 |
| Albumin | RHPEYAVSVLL | 1.63 | 0.40 | 233507 |
| Albumin | RHPEYAVSVLLR | 3.50 | 0.57 | 38508 |
| Albumin | RHPYFYAPE | 1.45 | 0.32 | 24598 |
| Albumin | RHPYFYAPEL | 1.67 | 0.25 | 48911 |
| Albumin | RHPYFYAPELL | 1.29 | 0.39 | 328315 |
| Albumin | RHPYFYAPELLY | 1.50 | 0.39 | 18409 |
| Albumin | RHPYFYAPELLYY | 1.53 | 0.36 | 74225 |
| Albumin | RHPYFYAPELLYYA | 1.92 | 0.42 | 29592 |
| Albumin | RHPYFYAPELLYYAN | 2.56 | 0.44 | 33033 |
| Albumin | RKVPQVSTPTLVEVSR | 2.28 | 0.59 | 6286 |
| Albumin | RPCFSALTPDETYVPK | 2.25 | 0.30 | 91968 |
| Albumin | SALTPDETYVPK | 1.60 | 0.40 | 1578848 |
| Albumin | SLHTLFGDEL | 1.62 | 0.38 | 4881 |
| Albumin | SLHTLFGDELC | 2.67 | 0.65 | 18403 |
| Albumin | SLHTLFGDELCK | 1.93 | 0.35 | 1545054 |
| Albumin | SQYLQQCPFDEHV | 2.85 | 0.64 | 2687 |
| Albumin | SQYLQQCPFDEHVK | 2.88 | 0.62 | 853416 |
| Albumin | TALVELLK | 1.80 | 0.63 | 5272 |
| Albumin | TFHADICTLPDTEK | 2.03 | 0.28 | 94648 |
| Albumin | TLCDEFKADEK | 1.51 | 0.53 | 5850 |
| Albumin | TLFGDELCK | 1.20 | 0.43 | 15789 |
| Albumin | TVMENFVA | 1.09 | 0.48 | 78665 |
| Albumin | TVMENFVAF | 1.25 | 0.44 | 17567 |
| Albumin | TVMENFVAFVDK | 0.91 | 0.40 | 996118 |
| Albumin | VDEPQNLIK | 2.02 | 0.60 | 5726 |
| Albumin | VPQVSTPTLVEVSR | 2.05 | 0.45 | 121050 |
| Albumin | YFYAPELL | 1.88 | 0.52 | 10251 |
| Albumin | YFYAPELLY | 2.08 | 0.28 | 47303 |
| Albumin | YFYAPELLYY | 3.18 | 0.62 | 8240 |
| Albumin | YFYAPELLYYANK | 2.97 | 0.55 | 4720 |
| Albumin | YICDNQDTISSK | 3.31 | 0.64 | 10665 |
| Albumin | YICDNQDTISSKLK | 1.49 | 0.34 | 1129 |
| Albumin | YLQQCPFDEHVK | 2.13 | 0.36 | 13121 |
| Albumin | YLYEIAR | 1.89 | 0.32 | 889663 |
| Albumin | YNGVFQECCQAEDK | 1.72 | 0.13 | 1348048 |
| Amylase | AAGVYCIGEVLDGDPAYTCPY | 1.92 | 0.68 | 12096 |
| Amylase | ASYTNDIALAK | 1.67 | 0.53 | 30879 |
| Amylase | ATPADWR | 1.04 | 0.30 | 164 |
| Amylase | CIGEVLDGDPAYTCPY | 2.15 | 0.44 | 9906 |
| Amylase | DFWPGYNK | 1.17 | 0.25 | 126934 |
| Amylase | DNTVSLPDLDTTK | 1.67 | 0.38 | 331059 |
| Amylase | DTGFVTYK | 1.30 | 0.49 | 8872 |
| Amylase | EATWLSGYPTDSELYK | 3.44 | 0.62 | 392568 |
| Amylase | ENYGTADDLK | 1.41 | 0.34 | 1102 |
| Amylase | FASYTNDIALAK | 2.64 | 0.40 | 170368 |
| Amylase | FIILNDGLPIIY | 2.11 | 0.50 | 2512 |
| Amylase | GSTTATCNTADQK | 1.32 | 0.34 | 767 |
| Amylase | GTDGSQIVTIL | 1.26 | 0.33 | 46549 |
| Amylase | GTDGSQIVTILSNK | 1.79 | 0.37 | 31034 |
| Amylase | GVLNYPIYYPL | 1.66 | 0.53 | 6377 |
| Amylase | GYDGAGSSVDYSVFKPF | 1.66 | 0.47 | 18513 |
| Amylase | GYPTDSELYK | 1.11 | 0.51 | 103774 |
| Amylase | HMGYDGAGSSVDY | 1.42 | 0.54 | 8344 |
| Amylase | HMGYDGAGSSVDYS | 1.73 | 0.68 | 3293 |
| Amylase | IDTVKHVQK | 1.06 | 0.43 | 5747 |
| Amylase | IYSLNENYGTADDLK | 1.63 | 0.47 | 90764 |
| Amylase | KGTDGSQIVTIL | 1.40 | 0.42 | 276849 |
| Amylase | KGTDGSQIVTILS | 1.34 | 0.46 | 25455 |
| Amylase | KGTDGSQIVTILSNK | 1.78 | 0.27 | 17001 |
| Amylase | LNENYGTADDLK | 2.08 | 0.46 | 1726 |
| Amylase | NDGLPIIYAGQEQHYAGGND | 3.50 | 0.76 | 36747 |
| Amylase | NDGLPIIYAGQEQHYAGGNDPANR | 1.55 | 0.35 | 544113 |
| Amylase | NEWYDWVGSL | 1.59 | 0.45 | 3308 |
| Amylase | NVMDGVLNYPIYYPL | 1.54 | 0.50 | 26594 |
| Amylase | NVMDGVLNYPIYYPLL | 1.83 | 0.47 | 6181 |
| Amylase | PDSTLLGTFVENHDNPR | 2.74 | 0.57 | 11376 |
| Amylase | QNVMDGVLNYPIYYPL | 2.50 | 0.56 | 10887 |
| Amylase | QNVMDGVLNYPIYYPLL | 2.26 | 0.48 | 2456 |
| Amylase | SDCPDSTLLGTF | 1.35 | 0.46 | 7046 |
| Amylase | SDCPDSTLLGTFVENHDNPR | 1.73 | 0.36 | 680300 |
| Amylase | SDGNVPVPMAGGLPR | 1.14 | 0.35 | 102505 |
| Amylase | SGSMDDLYNMINTVK | 3.02 | 0.55 | 2781 |
| Amylase | SGYPTDSELYK | 1.15 | 0.41 | 8396 |
| Amylase | SLNENYGTADDLK | 1.65 | 0.32 | 76178 |
| Amylase | SSQDYFHPF | 2.07 | 0.42 | 380804 |
| Amylase | STSGSMDDLYNMIN | 1.41 | 0.30 | 40183 |
| Amylase | STSGSMDDLYNMINTVK | 4.42 | 0.61 | 525764 |
| Amylase | SYTNDIALAK | 2.55 | 0.59 | 111989 |
| Amylase | TTVTVGSDGNVPVPMAGGLPR | 3.33 | 0.67 | 17391 |
| Amylase | TVTVGSDGNVPVPMAGGLPR | 3.12 | 0.62 | 23718 |
| Amylase | VDVVANHMGYDGAGSSVD | 2.96 | 0.62 | 6269 |
| Amylase | VDVVANHMGYDGAGSSVDYS | 4.17 | 0.64 | 12051 |
| Amylase | VGSDGNVPVPMAGGLPR | 2.39 | 0.57 | 40178 |
| Amylase | VLYPTEK | 1.23 | 0.46 | 745 |
| Amylase | VLYPTEKLAGSK | 1.07 | 0.49 | 3849 |
| Amylase | VTVGSDGNVPVPMAGGLPR | 2.76 | 0.62 | 8919 |
| Amylase | YCGGTWQGIIDK | 2.10 | 0.50 | 166613 |
| Amylase | YCGGTWQGIIDKL | 2.69 | 0.52 | 74617 |
| Amylase | YCGGTWQGIIDKLD | 2.19 | 0.38 | 81892 |
| Amylase | YCGGTWQGIIDKLDYI | 3.27 | 0.50 | 13173 |
| Amylase | YCGGTWQGIIDKLDYIQGM | 3.16 | 0.53 | 10414 |
| Catalase | AAQKPDVLTTGGGNPVGDK | 2.15 | 0.51 | 122130 |
| Catalase | AAQKPDVLTTGGGNPVGDKLN | 3.44 | 0.61 | 107817 |
| Catalase | AAQKPDVLTTGGGNPVGDKLNSL | 2.50 | 0.52 | 35706 |
| Catalase | AEIFPFNPFDLTK | 1.61 | 0.38 | 12688 |
| Catalase | AFDPSNMPPGIEPSPDK | 2.79 | 0.60 | 129547 |
| Catalase | AIATGNYPSWTL | 1.95 | 0.46 | 8118 |
| Catalase | AIATGNYPSWTLY | 1.82 | 0.62 | 24004 |
| Catalase | AQKPDVLTTGGGNPVGDK | 2.85 | 0.52 | 5730 |
| Catalase | AQKPDVLTTGGGNPVGDKLN | 2.42 | 0.52 | 1437752 |
| Catalase | DALLFPSFIH | 1.50 | 0.51 | 5923 |
| Catalase | DALLFPSFIHSQK | 1.30 | 0.26 | 25707 |
| Catalase | DAQLFIQK | 1.90 | 0.35 | 30405 |
| Catalase | DLFNAIATGNYPSWTL | 1.65 | 0.51 | 18575 |
| Catalase | DLFNAIATGNYPSWTLY | 2.03 | 0.52 | 2132 |
| Catalase | DMVWDFWSLRPESLHQVS | 2.14 | 0.57 | 3708 |
| Catalase | DPDMVWDFW | 1.37 | 0.46 | 953 |
| Catalase | DPDMVWDFWSLRPESLH | 3.54 | 0.60 | 4171 |
| Catalase | DVLTTGGGNPVGDKLN | 2.11 | 0.60 | 10151 |
| Catalase | DVVFTDEMAHFDR | 1.89 | 0.30 | 127291 |
| Catalase | EAEIFPFNPFDLTK | 1.55 | 0.28 | 8337 |
| Catalase | FNSANDDNVTQVR | 1.32 | 0.12 | 19709 |
| Catalase | FSEAEIFPFNPFDLTK | 2.82 | 0.50 | 10572 |
| Catalase | FSTVAGESGSADTVR | 2.72 | 0.48 | 14188 |
| Catalase | FSTVAGESGSADTVRD | 2.36 | 0.59 | 7797 |
| Catalase | FSTVAGESGSADTVRDPR | 1.23 | 0.39 | 157722 |
| Catalase | FYTEDGNWDLVGNNTPIF | 2.90 | 0.67 | 29575 |
| Catalase | GAGAFGYFEVT | 1.36 | 0.39 | 29527 |
| Catalase | GAGAFGYFEVTH | 1.31 | 0.41 | 64507 |
| Catalase | GAGAFGYFEVTHDITR | 2.25 | 0.43 | 482385 |
| Catalase | GNWDLVGNNTPIFFIR | 2.37 | 0.50 | 6615 |
| Catalase | GPLLVQDVVFTDEMAHF | 3.87 | 0.72 | 4308 |
| Catalase | GPLLVQDVVFTDEMAHFD | 4.55 | 0.51 | 7948 |
| Catalase | GPLLVQDVVFTDEMAHFDR | 3.35 | 0.49 | 370088 |
| Catalase | GYFEVTHDITR | 1.54 | 0.30 | 83233 |
| Catalase | IQALLDKYNEEKPK | 1.99 | 0.32 | 180641 |
| Catalase | IQALLDKYNEEKPKN | 1.95 | 0.31 | 43245 |
| Catalase | KDPDMVWDFW | 1.92 | 0.60 | 3976 |
| Catalase | LAHEDPDYGLR | 2.38 | 0.60 | 778 |
| Catalase | LCENIAGHLK | 1.07 | 0.46 | 57167 |
| Catalase | LFAYPDTHR | 1.46 | 0.42 | 14842 |
| Catalase | LGPNYLQIPVN | 1.01 | 0.43 | 43802 |
| Catalase | LGPNYLQIPVNCPYR | 2.35 | 0.26 | 1006461 |
| Catalase | LGPNYLQIPVNCPYRA | 3.22 | 0.67 | 44984 |
| Catalase | LKDPDMVWDFW | 2.17 | 0.49 | 21699 |
| Catalase | LLDKYNEEKPKN | 2.50 | 0.40 | 9932 |
| Catalase | LLVQDVVFTDEMAHFDR | 3.13 | 0.54 | 41847 |
| Catalase | LQIPVNCPYR | 1.90 | 0.58 | 49123 |
| Catalase | LVNADGEAVYCK | 2.53 | 0.49 | 5873 |
| Catalase | MTFSEAEIFPFNPFDLTK | 3.07 | 0.44 | 35355 |
| Catalase | NFSDVHPEY | 1.54 | 0.33 | 5247 |
| Catalase | NFSDVHPEYGSR | 1.30 | 0.22 | 14893 |
| Catalase | NLSVEDAAR | 1.61 | 0.50 | 1916 |
| Catalase | NPVNYFAEVEQL | 1.88 | 0.24 | 57927 |
| Catalase | NPVNYFAEVEQLAFD | 1.54 | 0.47 | 18694 |
| Catalase | NPVNYFAEVEQLAFDPS | 1.89 | 0.58 | 2431 |
| Catalase | PDMVWDFWSLRPESLHQVS | 2.59 | 0.41 | 7841 |
| Catalase | PHGDYPLIPVGK | 1.59 | 0.44 | 86771 |
| Catalase | PNSFSAPEHQPSALEHR | 1.78 | 0.42 | 14136 |
| Catalase | PSNMPPGIEPSPDK | 1.75 | 0.44 | 44017 |
| Catalase | QDVVFTDEMAHFDR | 1.95 | 0.31 | 21611 |
| Catalase | QKPDVLTTGGGNPVGDK | 1.91 | 0.48 | 2526 |
| Catalase | QKPDVLTTGGGNPVGDKLN | 2.01 | 0.50 | 11125 |
| Catalase | QVSFLFSDR | 1.61 | 0.59 | 1134 |
| Catalase | RNPQTHLKDPDMVWDFW | 3.05 | 0.63 | 38748 |
| Catalase | SAPEHQPSALEHR | 2.52 | 0.73 | 747 |
| Catalase | SEAEIFPFNPFDLT | 1.74 | 0.52 | 2238 |
| Catalase | SEAEIFPFNPFDLTK | 1.93 | 0.51 | 150882 |
| Catalase | SFSAPEHQPSAL | 1.56 | 0.39 | 18564 |
| Catalase | SFSAPEHQPSALE | 1.62 | 0.48 | 6572 |
| Catalase | SFSAPEHQPSALEHR | 1.79 | 0.45 | 4624 |
| Catalase | SLRPESLHQVS | 1.63 | 0.47 | 229 |
| Catalase | SLRPESLHQVSF | 1.29 | 0.48 | 26139 |
| Catalase | TEDGNWDLVGNNTPIFFIR | 3.11 | 0.65 | 3352 |
| Catalase | TFSEAEIFPFNPF | 1.83 | 0.48 | 4134 |
| Catalase | TFSEAEIFPFNPFDLTK | 3.10 | 0.60 | 468660 |
| Catalase | VGNNTPIFFIR | 1.98 | 0.53 | 9538 |
| Catalase | VVFTDEMAHFDR | 2.13 | 0.55 | 411 |
| Catalase | VWPHGDYPLIPVGK | 2.05 | 0.46 | 1272823 |
| Catalase | YLQIPVNCPYR | 1.47 | 0.34 | 61142 |
| Catalase | YTEDGNWDLVGNNTPIFFIR | 3.73 | 0.65 | 2492 |
| Lactoglobulin | AASDISLLDAQSAPLR | 2.01 | 0.23 | 155103 |
| Lactoglobulin | AMAASDISLLDAQSAPL | 3.27 | 0.52 | 38621 |
| Lactoglobulin | AMAASDISLLDAQSAPLR | 1.59 | 0.35 | 1187961 |
| Lactoglobulin | ASDISLLDAQSAPLR | 1.99 | 0.48 | 823961 |
| Lactoglobulin | CMENSAEPEQSLVC | 1.58 | 0.44 | 34998 |
| Lactoglobulin | CMENSAEPEQSLVCQ | 2.78 | 0.45 | 179325 |
| Lactoglobulin | CMENSAEPEQSLVCQCL | 3.09 | 0.46 | 1237 |
| Lactoglobulin | DISLLDAQSAPLR | 1.79 | 0.18 | 44573 |
| Lactoglobulin | EELKPTPEGDLEILLQ | 3.04 | 0.41 | 16622 |
| Lactoglobulin | EELKPTPEGDLEILLQK | 2.78 | 0.49 | 12372 |
| Lactoglobulin | ELKPTPEGDLEIL | 1.77 | 0.36 | 156297 |
| Lactoglobulin | ELKPTPEGDLEILL | 2.25 | 0.38 | 93726 |
| Lactoglobulin | ELKPTPEGDLEILLQ | 2.39 | 0.38 | 106620 |
| Lactoglobulin | ELKPTPEGDLEILLQK | 2.52 | 0.24 | 39575 |
| Lactoglobulin | ENSAEPEQSLVCQ | 1.65 | 0.45 | 20886 |
| Lactoglobulin | EVDDEALEKFDK | 1.57 | 0.41 | 626997 |
| Lactoglobulin | FNPTQLEEQCHI | 2.80 | 0.58 | 3480273 |
| Lactoglobulin | IDALNENKVL | 2.10 | 0.37 | 38947 |
| Lactoglobulin | KPTPEGDLEILLQK | 1.61 | 0.23 | 15351 |
| Lactoglobulin | KVAGTWY | 1.84 | 0.59 | 5475 |
| Lactoglobulin | LIVTQTMK | 1.56 | 0.22 | 4131 |
| Lactoglobulin | LKPTPEGDLEIL | 1.74 | 0.38 | 12837 |
| Lactoglobulin | LSFNPTQLEEQCHI | 1.81 | 0.30 | 4852920 |
| Lactoglobulin | LVLDTDYK | 1.08 | 0.52 | 1762 |
| Lactoglobulin | LVLDTDYKK | 1.77 | 0.22 | 28252 |
| Lactoglobulin | MAASDISLLDAQSAPL | 2.85 | 0.58 | 190755 |
| Lactoglobulin | MAASDISLLDAQSAPLR | 1.80 | 0.34 | 3218162 |
| Lactoglobulin | NPTQLEEQCHI | 2.38 | 0.53 | 23105 |
| Lactoglobulin | PEVDDEALEKFDK | 1.70 | 0.41 | 23809 |
| Lactoglobulin | PTQLEEQCHI | 1.50 | 0.44 | 2115 |
| Lactoglobulin | QLEEQCHI | 1.24 | 0.45 | 973 |
| Lactoglobulin | SDISLLDAQSAPLR | 1.64 | 0.39 | 54077 |
| Lactoglobulin | SFNPTQLEEQCHI | 1.50 | 0.37 | 107454 |
| Lactoglobulin | SLAMAASDISLL | 1.27 | 0.55 | 10257 |
| Lactoglobulin | SLAMAASDISLLDAQSAPLR | 2.59 | 0.61 | 163244 |
| Lactoglobulin | TKIPAVFK | 1.69 | 0.37 | 253739 |
| Lactoglobulin | TKIPAVFKID | 2.11 | 0.54 | 8112 |
| Lactoglobulin | TPEVDDEALEK | 2.98 | 0.63 | 64192 |
| Lactoglobulin | TPEVDDEALEKF | 2.19 | 0.55 | 49006 |
| Lactoglobulin | TPEVDDEALEKFD | 2.47 | 0.58 | 82454 |
| Lactoglobulin | TPEVDDEALEKFDK | 2.64 | 0.53 | 4864823 |
| Lactoglobulin | TPEVDDEALEKFDKA | 2.96 | 0.52 | 60801 |
| Lactoglobulin | VEELKPTPEGDLEIL | 2.97 | 0.49 | 513091 |
| Lactoglobulin | VEELKPTPEGDLEILL | 2.88 | 0.27 | 397925 |
| Lactoglobulin | VEELKPTPEGDLEILLQ | 3.31 | 0.50 | 486103 |
| Lactoglobulin | VEELKPTPEGDLEILLQK | 4.07 | 0.46 | 16457 |
| Lactoglobulin | VLDTDYKK | 1.91 | 0.65 | 26667 |
| Lactoglobulin | VLVLDTDYK | 2.36 | 0.51 | 1489775 |
| Lactoglobulin | VLVLDTDYKK | 2.51 | 0.52 | 1303144 |
| Lactoglobulin | VRTPEVDDEALEK | 2.41 | 0.44 | 18008 |
| Lactoglobulin | VRTPEVDDEALEKFDK | 4.52 | 0.60 | 20385 |
| Lactoglobulin | VYVEELKPTPEGD | 1.57 | 0.51 | 25639 |
| Lactoglobulin | VYVEELKPTPEGDLEIL | 1.66 | 0.31 | 2559724 |
| Lactoglobulin | VYVEELKPTPEGDLEILL | 2.53 | 0.49 | 2403676 |
| Lactoglobulin | VYVEELKPTPEGDLEILLQ | 2.77 | 0.58 | 2675863 |
| Lactoglobulin | VYVEELKPTPEGDLEILLQK | 4.25 | 0.62 | 3560642 |
| Myoglobin | ADIAGHGQEVLIR | 1.57 | 0.32 | 111827 |
| Myoglobin | ALELFRNDIAAK | 1.49 | 0.43 | 402941 |
| Myoglobin | ALGGILKK | 2.19 | 0.64 | 190 |
| Myoglobin | ASEDLKKHGTVVLTALGGILK | 4.24 | 0.61 | 29586 |
| Myoglobin | DIAGHGQEVLIR | 1.52 | 0.35 | 18923 |
| Myoglobin | EADIAGHGQEVLIR | 1.87 | 0.43 | 60012 |
| Myoglobin | EFISDAIIH | 1.39 | 0.51 | 13352 |
| Myoglobin | EFISDAIIHVL | 1.77 | 0.44 | 26679 |
| Myoglobin | ELFRNDIAAK | 2.02 | 0.58 | 8610 |
| Myoglobin | FISDAIIH | 1.48 | 0.49 | 10132 |
| Myoglobin | FISDAIIHVL | 1.91 | 0.56 | 19292 |
| Myoglobin | FISDAIIHVLH | 2.38 | 0.45 | 6100 |
| Myoglobin | FTGHPETLEK | 2.17 | 0.59 | 15492 |
| Myoglobin | GEWQQVLNVWGK | 1.42 | 0.54 | 5121 |
| Myoglobin | GHPETLEKFDK | 1.52 | 0.44 | 5701 |
| Myoglobin | GKVEADIAGHGQEVLIR | 3.37 | 0.56 | 29925 |
| Myoglobin | GLSDGEWQQVL | 2.06 | 0.50 | 975113 |
| Myoglobin | GLSDGEWQQVLN | 1.64 | 0.39 | 171973 |
| Myoglobin | GLSDGEWQQVLNVW | 1.44 | 0.46 | 35731 |
| Myoglobin | GLSDGEWQQVLNVWGK | 3.33 | 0.61 | 1588911 |
| Myoglobin | GNFGADAQGAMTK | 1.35 | 0.41 | 431853 |
| Myoglobin | HGQEVLIR | 1.53 | 0.58 | 3976 |
| Myoglobin | HGTVVLTAL | 1.15 | 0.43 | 7395 |
| Myoglobin | HGTVVLTALGGILK | 2.97 | 0.52 | 498263 |
| Myoglobin | HGTVVLTALGGILKK | 3.44 | 0.47 | 252835 |
| Myoglobin | HKIPIKYLEFISDAIIHVL | 4.14 | 0.70 | 6247 |
| Myoglobin | HPGNFGADAQGAMT | 1.63 | 0.44 | 3856 |
| Myoglobin | HPGNFGADAQGAMTK | 1.51 | 0.35 | 43738 |
| Myoglobin | IAGHGQEVLIR | 1.91 | 0.55 | 2334 |
| Myoglobin | ISDAIIHVL | 3.05 | 0.72 | 17632 |
| Myoglobin | LEFISDAIIH | 1.54 | 0.57 | 761471 |
| Myoglobin | LEFISDAIIHV | 1.64 | 0.42 | 19701 |
| Myoglobin | LEFISDAIIHVL | 2.00 | 0.40 | 1282117 |
| Myoglobin | LEFISDAIIHVLH | 2.30 | 0.44 | 1176790 |
| Myoglobin | LEFISDAIIHVLHSK | 1.68 | 0.42 | 99546 |
| Myoglobin | LFTGHPETL | 1.54 | 0.40 | 73362 |
| Myoglobin | LFTGHPETLEK | 1.45 | 0.23 | 544072 |
| Myoglobin | LFTGHPETLEKFDK | 1.83 | 0.45 | 148015 |
| Myoglobin | RLFTGHPETLEK | 2.77 | 0.58 | 7190 |
| Myoglobin | SDGEWQQVLNVWGK | 1.89 | 0.20 | 337260 |
| Myoglobin | SKHPGNFGADAQGAMTK | 2.99 | 0.48 | 2038 |
| Myoglobin | TGHPETLEK | 1.75 | 0.45 | 9305 |
| Myoglobin | VEADIAGHGQEVLI | 1.67 | 0.33 | 487076 |
| Myoglobin | VEADIAGHGQEVLIR | 1.47 | 0.30 | 6013115 |
| Myoglobin | YKELGFQ | 1.64 | 0.44 | 8335 |
| Myoglobin | YKELGFQG | 2.01 | 0.50 | 878981 |
| Myoglobin | YLEFISDAIIH | 1.90 | 0.44 | 113655 |
| Myoglobin | YLEFISDAIIHV | 1.85 | 0.45 | 4529 |
| Myoglobin | YLEFISDAIIHVL | 1.56 | 0.49 | 426685 |
| Myoglobin | YLEFISDAIIHVLH | 2.30 | 0.52 | 311454 |
| Myoglobin | YLEFISDAIIHVLHSK | 1.82 | 0.35 | 246538 |
| Ovalbumin | ADHPFLF | 1.23 | 0.39 | 202195 |
| Ovalbumin | ADHPFLFCIK | 2.61 | 0.54 | 14197 |
| Ovalbumin | AEERYPILPEYLQCVK | 2.18 | 0.43 | 17163 |
| Ovalbumin | ALAMVYLGAK | 2.32 | 0.55 | 201528 |
| Ovalbumin | ALAMVYLGAKDSTR | 2.11 | 0.56 | 113883 |
| Ovalbumin | AMGITDVFSSSANLSGISSAESLK | 2.38 | 0.45 | 90123 |
| Ovalbumin | ANLSGISSAESLK | 1.98 | 0.55 | 6732 |
| Ovalbumin | AVLFFGR | 1.47 | 0.68 | 4785 |
| Ovalbumin | DEDTQAMPFR | 1.05 | 0.22 | 61740 |
| Ovalbumin | DEVSGLEQLESIINFEK | 2.56 | 0.43 | 1984 |
| Ovalbumin | DILNQITK | 1.70 | 0.40 | 46315 |
| Ovalbumin | DILNQITKPNDVY | 1.43 | 0.41 | 18888 |
| Ovalbumin | DILNQITKPNDVYS | 1.59 | 0.33 | 12257 |
| Ovalbumin | DILNQITKPNDVYSF | 2.60 | 0.60 | 441277 |
| Ovalbumin | DILNQITKPNDVYSFS | 1.71 | 0.52 | 25750 |
| Ovalbumin | DILNQITKPNDVYSFSL | 1.78 | 0.30 | 8189 |
| Ovalbumin | DILNQITKPNDVYSFSLA | 2.32 | 0.59 | 316407 |
| Ovalbumin | DVFSSSANLSGISSAESLK | 2.95 | 0.63 | 17237 |
| Ovalbumin | EKLTEWTSSNVMEER | 3.42 | 0.57 | 56123 |
| Ovalbumin | ELINSWVESQTNGIIR | 1.58 | 0.37 | 1513861 |
| Ovalbumin | ELPFASGTMSML | 1.60 | 0.51 | 23454 |
| Ovalbumin | ELYRGGLEPINFQTAADQAR | 4.27 | 0.56 | 24227 |
| Ovalbumin | ESIINFEK | 1.63 | 0.46 | 10814 |
| Ovalbumin | EVVGSAEAGVDAASVSEEFR | 4.32 | 0.75 | 60324 |
| Ovalbumin | GGLEPINFQTA | 1.21 | 0.28 | 48969 |
| Ovalbumin | GGLEPINFQTAADQAR | 4.13 | 0.68 | 736538 |
| Ovalbumin | GITDVFSSSANLSGISSAESLK | 3.82 | 0.70 | 42241 |
| Ovalbumin | HIATNAVLFFGR | 1.37 | 0.34 | 901750 |
| Ovalbumin | IKVYLPR | 1.46 | 0.33 | 14856 |
| Ovalbumin | ILELPFASGTM | 1.34 | 0.55 | 113164 |
| Ovalbumin | ILELPFASGTMS | 1.91 | 0.37 | 30723 |
| Ovalbumin | ILELPFASGTMSM | 1.60 | 0.37 | 9804 |
| Ovalbumin | ILELPFASGTMSML | 2.20 | 0.49 | 265242 |
| Ovalbumin | ILPEYLQ | 1.60 | 0.36 | 15435 |
| Ovalbumin | ILPEYLQCVK | 1.50 | 0.39 | 582598 |
| Ovalbumin | ISQAVHAAHAEINEAGR | 4.69 | 0.68 | 964 |
| Ovalbumin | LPDEVSGLEQLESIINFEK | 3.35 | 0.66 | 9561 |
| Ovalbumin | LTEWTSSNVM | 1.35 | 0.49 | 17588 |
| Ovalbumin | LTEWTSSNVMEER | 2.07 | 0.41 | 1036618 |
| Ovalbumin | LTEWTSSNVMEERK | 2.12 | 0.43 | 30481 |
| Ovalbumin | LVLLPDEVSGLEQLE | 1.87 | 0.42 | 1899 |
| Ovalbumin | LYAEERYPILPEYLQCVK | 2.82 | 0.51 | 574521 |
| Ovalbumin | MKILELPFASGTM | 2.02 | 0.50 | 1828 |
| Ovalbumin | MKILELPFASGTMSML | 3.83 | 0.69 | 16974 |
| Ovalbumin | MMYQIGLFR | 1.39 | 0.50 | 1869 |
| Ovalbumin | MVLVNAIVFK | 2.53 | 0.71 | 6497 |
| Ovalbumin | NVLQPSSVDSQTA | 1.33 | 0.38 | 44845 |
| Ovalbumin | NVLQPSSVDSQTAMVL | 1.41 | 0.45 | 226699 |
| Ovalbumin | NVLQPSSVDSQTAMVLV | 1.92 | 0.37 | 69463 |
| Ovalbumin | NVLQPSSVDSQTAMVLVN | 1.96 | 0.30 | 184974 |
| Ovalbumin | PSSVDSQTAMVLVNAIVFK | 1.90 | 0.49 | 71725 |
| Ovalbumin | QITKPNDVYSFSLA | 2.44 | 0.62 | 38288 |
| Ovalbumin | QITKPNDVYSFSLASR | 2.12 | 0.37 | 22583 |
| Ovalbumin | SALAMVYLGAK | 1.63 | 0.45 | 27891 |
| Ovalbumin | SANLSGISSAESLK | 1.46 | 0.33 | 8489 |
| Ovalbumin | SIINFEK | 1.14 | 0.35 | 15371 |
| Ovalbumin | SMEFCFDVFK | 1.34 | 0.49 | 8810 |
| Ovalbumin | SQTAMVLVNAIVFK | 1.87 | 0.36 | 2155 |
| Ovalbumin | SSSANLSGISSAESLK | 1.80 | 0.36 | 31803 |
| Ovalbumin | SVDSQTAMVLVNAIVFK | 2.12 | 0.51 | 1461 |
| Ovalbumin | SVSEEFRADHPFLFCIK | 3.35 | 0.62 | 33625 |
| Ovalbumin | SWVESQTNGIIR | 2.06 | 0.10 | 26112 |
| Ovalbumin | VHHANENIFY | 1.46 | 0.39 | 13393 |
| Ovalbumin | VHHANENIFYCPIAIM | 1.42 | 0.40 | 166174 |
| Ovalbumin | VHHANENIFYCPIAIMS | 2.40 | 0.51 | 41131 |
| Ovalbumin | VHHANENIFYCPIAIMSAL | 1.95 | 0.43 | 2570 |
| Ovalbumin | VLLPDEVSGLEQL | 2.37 | 0.48 | 8721 |
| Ovalbumin | VLLPDEVSGLEQLESIIN | 2.40 | 0.41 | 46945 |
| Ovalbumin | VLLPDEVSGLEQLESIINFEK | 2.43 | 0.51 | 461137 |
| Ovalbumin | VTEQESKPVQMM | 1.56 | 0.43 | 90297 |
| Ovalbumin | VTEQESKPVQMMY | 2.00 | 0.54 | 291018 |
| Ovalbumin | YPILPEYLQ | 1.00 | 0.37 | 9950 |
| Ovalbumin | YPILPEYLQCVK | 1.89 | 0.28 | 479803 |
| Ovalbumin | YQIGLFR | 1.94 | 0.77 | 293264 |
| Transferrin | AAANFFSASCVPCADQSSFPK | 2.26 | 0.39 | 186451 |
| Transferrin | ACAVLGLCLADPER | 1.34 | 0.45 | 36319 |
| Transferrin | ADAMSLDGGYLYIAGK | 1.53 | 0.39 | 9000 |
| Transferrin | AENCHLAR | 0.93 | 0.57 | 10155 |
| Transferrin | AIECETAENTEECIAK | 3.29 | 0.07 | 20558 |
| Transferrin | AISNNEADAVTLDGGLVY | 1.95 | 0.34 | 29225 |
| Transferrin | AISNNEADAVTLDGGLVYEA | 1.61 | 0.47 | 3797 |
| Transferrin | AISNNEADAVTLDGGLVYEAGLK | 1.59 | 0.33 | 2106 |
| Transferrin | AISNNEADAVTLDGGLVYEAGLKP | 2.00 | 0.38 | 60786 |
| Transferrin | ALCIGSEK | 2.44 | 0.59 | 512 |
| Transferrin | AMSLDGGYLYIAGK | 1.76 | 0.44 | 4866 |
| Transferrin | ANFFSASCVPCADQSSFPK | 4.16 | 0.66 | 37481 |
| Transferrin | ASCVPCADQSSFPK | 1.78 | 0.34 | 11412 |
| Transferrin | CACSNHEPYFGYS | 1.21 | 0.45 | 2875 |
| Transferrin | CACSNHEPYFGYSGAFK | 3.14 | 0.44 | 47289 |
| Transferrin | CAVLGLCLADP | 1.48 | 0.46 | 32074 |
| Transferrin | CGLVPVLAENYK | 0.97 | 0.33 | 1108309 |
| Transferrin | CKFDEFFSAGCAPGSPR | 2.68 | 0.37 | 18615 |
| Transferrin | CLMEGAGDVAFVK | 1.61 | 0.43 | 211082 |
| Transferrin | CLMEGAGDVAFVKH | 1.85 | 0.44 | 37566 |
| Transferrin | CLMEGAGDVAFVKHSTVF | 2.05 | 0.49 | 2851 |
| Transferrin | CSNHEPYFGY | 1.36 | 0.52 | 8058 |
| Transferrin | CSNHEPYFGYS | 1.77 | 0.53 | 12057 |
| Transferrin | CSNHEPYFGYSGAFK | 2.40 | 0.37 | 368640 |
| Transferrin | CVPCADQSSFPK | 2.23 | 0.48 | 339138 |
| Transferrin | CVPCADQSSFPKL | 2.75 | 0.52 | 67001 |
| Transferrin | DANINWNNLK | 1.75 | 0.48 | 7436 |
| Transferrin | DEFFSAGCAPGSPR | 2.04 | 0.02 | 15327 |
| Transferrin | DGGLVYEAGLKPNNLKPVVA | 2.79 | 0.59 | 864 |
| Transferrin | DKPDNFQLF | 2.08 | 0.27 | 1086168 |
| Transferrin | DKPDNFQLFQSPHGK | 2.44 | 0.48 | 5098 |
| Transferrin | DLLFRDDTK | 1.73 | 0.40 | 40728 |
| Transferrin | DNPQTHYY | 2.07 | 0.66 | 912 |
| Transferrin | DNPQTHYYAV | 1.91 | 0.45 | 27085 |
| Transferrin | DNPQTHYYAVA | 1.41 | 0.45 | 378586 |
| Transferrin | DNPQTHYYAVAVVK | 1.81 | 0.46 | 546967 |
| Transferrin | DQTVIQNTDGNNNEAWAK | 2.12 | 0.41 | 1872192 |
| Transferrin | DQTVIQNTDGNNNEAWAKNLK | 2.18 | 0.44 | 9460 |
| Transferrin | DSADGFLK | 1.39 | 0.27 | 9603 |
| Transferrin | EDVIWELL | 1.45 | 0.21 | 64373 |
| Transferrin | EDVIWELLNHAQ | 1.93 | 0.36 | 1216 |
| Transferrin | EDVIWELLNHAQEHFGK | 2.53 | 0.55 | 40672 |
| Transferrin | EFFSAGCAPGSPR | 1.76 | 0.30 | 3328 |
| Transferrin | ELLCGDNTRK | 2.18 | 0.49 | 4592 |
| Transferrin | ELPDPQESIQR | 1.59 | 0.57 | 2269778 |
| Transferrin | ENFEVLCK | 1.26 | 0.34 | 289202 |
| Transferrin | ESGPFVSCVK | 1.41 | 0.36 | 82638 |
| Transferrin | FDEFFSAGCAPG | 1.47 | 0.35 | 8116 |
| Transferrin | FDEFFSAGCAPGSPR | 1.50 | 0.25 | 3340319 |
| Transferrin | FFSAGCAPGSPR | 2.16 | 0.62 | 5467 |
| Transferrin | FGYSGAFK | 1.97 | 0.58 | 7630 |
| Transferrin | FKDSADGFLK | 1.97 | 0.47 | 3828 |
| Transferrin | FSAGCAPGSPR | 1.99 | 0.50 | 9078 |
| Transferrin | FSASCVPCADQSSFPK | 1.63 | 0.47 | 10326 |
| Transferrin | FSGGAIECETAENTEECIAK | 4.33 | 0.38 | 5823 |
| Transferrin | FVKDQTVIQNTDGNNNEAWAK | 3.74 | 0.54 | 89125 |
| Transferrin | GAIECETAENTEECIAK | 3.13 | 0.10 | 4859 |
| Transferrin | GDVAFVKDQTVIQ | 2.06 | 0.40 | 8656 |
| Transferrin | GEADAMSLDGGYLY | 1.69 | 0.30 | 95318 |
| Transferrin | GEADAMSLDGGYLYIA | 1.47 | 0.33 | 248835 |
| Transferrin | GEADAMSLDGGYLYIAGK | 2.70 | 0.38 | 215341 |
| Transferrin | GLKPNNLKPVVAEFHGTK | 1.91 | 0.37 | 6648 |
| Transferrin | GLVPVLAENYK | 1.30 | 0.44 | 10186 |
| Transferrin | GRSAGWNIPMAKLYK | 1.78 | 0.51 | 3491 |
| Transferrin | GYLAVAVVK | 2.02 | 0.41 | 40118 |
| Transferrin | HSTVFDNLPNPEDR | 2.25 | 0.46 | 1396683 |
| Transferrin | HSTVFDNLPNPEDRK | 2.49 | 0.47 | 936168 |
| Transferrin | ILESGPFVS | 1.29 | 0.36 | 17075 |
| Transferrin | ILESGPFVSCVK | 1.91 | 0.56 | 403408 |
| Transferrin | IMKGEADAMSLD | 1.86 | 0.43 | 57942 |
| Transferrin | IMKGEADAMSLDGGYLY | 3.76 | 0.60 | 128915 |
| Transferrin | IMKGEADAMSLDGGYLYIA | 2.43 | 0.48 | 110527 |
| Transferrin | KELPDPQESIQ | 2.09 | 0.53 | 63184 |
| Transferrin | KELPDPQESIQR | 2.48 | 0.52 | 15806 |
| Transferrin | KENFEVL | 1.33 | 0.35 | 78355 |
| Transferrin | KENFEVLCK | 2.05 | 0.53 | 3427 |
| Transferrin | KGEADAMSLDGGYLY | 2.20 | 0.59 | 69433 |
| Transferrin | KGEADAMSLDGGYLYIA | 2.67 | 0.59 | 280141 |
| Transferrin | KGEADAMSLDGGYLYIAGK | 3.53 | 0.63 | 26899 |
| Transferrin | KLLEACTFHKP | 2.91 | 0.41 | 4056 |
| Transferrin | KNYELLCGDNTR | 2.88 | 0.04 | 19515 |
| Transferrin | KSVDDYQECYL | 1.28 | 0.31 | 26750 |
| Transferrin | KSVDDYQECYLA | 2.14 | 0.50 | 511535 |
| Transferrin | KTYDSYLGDDYV | 1.41 | 0.40 | 9767 |
| Transferrin | KTYDSYLGDDYVR | 1.94 | 0.27 | 425264 |
| Transferrin | LGYEYVTALQ | 2.78 | 0.57 | 110726 |
| Transferrin | LLEACTFHKP | 1.19 | 0.34 | 423165 |
| Transferrin | LMEGAGDVAFVK | 2.51 | 0.65 | 19230 |
| Transferrin | LVPVLAENYK | 1.51 | 0.44 | 32112 |
| Transferrin | LYKELPDPQESIQR | 2.07 | 0.41 | 59147 |
| Transferrin | MDFELYLGYEYVT | 1.96 | 0.52 | 806 |
| Transferrin | MDFELYLGYEYVTA | 1.70 | 0.44 | 3103 |
| Transferrin | MDFELYLGYEYVTAL | 1.55 | 0.30 | 46881 |
| Transferrin | MDFELYLGYEYVTALQ | 1.32 | 0.36 | 113554 |
| Transferrin | MDFELYLGYEYVTALQNL | 3.18 | 0.50 | 610 |
| Transferrin | MEGAGDVAFVK | 1.32 | 0.29 | 613991 |
| Transferrin | MEGAGDVAFVKH | 2.07 | 0.54 | 121402 |
| Transferrin | MVPSHAVVA | 1.23 | 0.61 | 362 |
| Transferrin | NHEPYFGY | 1.59 | 0.73 | 5320 |
| Transferrin | NHEPYFGYSGA | 1.58 | 0.51 | 3111 |
| Transferrin | NHEPYFGYSGAFK | 2.62 | 0.55 | 220383 |
| Transferrin | NLKPVVAEFHGTK | 3.03 | 0.42 | 52047 |
| Transferrin | NNLKPVVAEFHGTK | 2.36 | 0.38 | 50194 |
| Transferrin | NTPEKGYLAVAVVK | 1.64 | 0.29 | 7460 |
| Transferrin | NYELLCGDNTR | 3.05 | 0.47 | 789750 |
| Transferrin | NYELLCGDNTRK | 1.86 | 0.33 | 31644 |
| Transferrin | PCADQSSFPK | 1.47 | 0.40 | 2654 |
| Transferrin | PDPQESIQR | 1.34 | 0.32 | 21245 |
| Transferrin | PVLAENYK | 2.26 | 0.73 | 4895 |
| Transferrin | QTHYYAVAVVKK | 1.22 | 0.25 | 2717 |
| Transferrin | QTVIQNTDGNNNEAWAK | 3.29 | 0.10 | 36331 |
| Transferrin | RGPNHAVVSRK | 1.54 | 0.46 | 1697 |
| Transferrin | RTVGGKEDVIWELL | 1.98 | 0.51 | 109932 |
| Transferrin | SAGWNIPM | 1.85 | 0.54 | 21810 |
| Transferrin | SASCVPCADQSSFPK | 2.25 | 0.48 | 78627 |
| Transferrin | SASCVPCADQSSFPKL | 2.60 | 0.48 | 6735 |
| Transferrin | SCVPCADQSSFPK | 2.98 | 0.54 | 616395 |
| Transferrin | SCVPCADQSSFPKL | 2.57 | 0.55 | 46402 |
| Transferrin | SGGAIECETAENTEECIAK | 4.03 | 0.67 | 96253 |
| Transferrin | SKDLLFR | 1.88 | 0.45 | 623 |
| Transferrin | SKMDFELY | 2.36 | 0.61 | 9411 |
| Transferrin | SKMDFELYLGYEYVTALQ | 2.56 | 0.62 | 17224 |
| Transferrin | SLDGGYLYIAGK | 2.72 | 0.58 | 22283 |
| Transferrin | SNHEPYFGYSGAFK | 2.73 | 0.56 | 24159 |
| Transferrin | STVFDNLPNPEDR | 1.73 | 0.45 | 770154 |
| Transferrin | STVFDNLPNPEDRK | 1.86 | 0.41 | 302765 |
| Transferrin | SVDDYQECY | 1.54 | 0.35 | 3928 |
| Transferrin | SVDDYQECYL | 1.78 | 0.44 | 26669 |
| Transferrin | SVDDYQECYLA | 1.63 | 0.31 | 295770 |
| Transferrin | SVDDYQECYLAMVPSHAVVA | 1.81 | 0.51 | 6717 |
| Transferrin | SVTDCTSNF | 1.45 | 0.41 | 16725 |
| Transferrin | SVTDCTSNFC | 1.47 | 0.44 | 12499 |
| Transferrin | SVTDCTSNFCL | 1.37 | 0.27 | 17690 |
| Transferrin | SVTDCTSNFCLF | 1.52 | 0.40 | 1004640 |
| Transferrin | SYLGDDYVR | 1.25 | 0.29 | 30484 |
| Transferrin | TAGWNIPM | 2.24 | 0.38 | 156861 |
| Transferrin | TAGWNIPMGLL | 1.27 | 0.39 | 154313 |
| Transferrin | TAGWNIPMGLLYSK | 2.00 | 0.51 | 32772 |
| Transferrin | TSDANINW | 1.15 | 0.44 | 36884 |
| Transferrin | TSDANINWNNLK | 3.04 | 0.44 | 616418 |
| Transferrin | TSDANINWNNLKDK | 1.59 | 0.38 | 31761 |
| Transferrin | TSDANINWNNLKDKK | 1.53 | 0.35 | 11040 |
| Transferrin | TVFDNLPNPEDR | 1.62 | 0.36 | 12739 |
| Transferrin | TVGGKEDVIWE | 1.79 | 0.41 | 5545 |
| Transferrin | TVGGKEDVIWELL | 2.22 | 0.47 | 1893697 |
| Transferrin | TVIQNTDGNNNEAWAK | 3.57 | 0.74 | 4452 |
| Transferrin | TYDSYLGDDYV | 1.83 | 0.59 | 13547 |
| Transferrin | TYDSYLGDDYVR | 2.94 | 0.66 | 2100033 |
| Transferrin | TYDSYLGDDYVRA | 1.84 | 0.50 | 4446 |
| Transferrin | VIWELLNHAQEH | 1.77 | 0.58 | 595 |
| Transferrin | VKTSDANINWNNLK | 1.94 | 0.44 | 63216 |
| Transferrin | VLRILESGPFVSCV | 2.41 | 0.64 | 23715 |
| Transferrin | VTDAENCHLA | 1.33 | 0.57 | 4124 |
| Transferrin | VTDAENCHLAR | 1.43 | 0.46 | 19282 |
| Transferrin | WCTISTHEANK | 1.36 | 0.30 | 248 |
| Transferrin | WELLNHAQEHFGK | 2.38 | 0.51 | 15565 |
| Transferrin | YELLCGDNTR | 1.29 | 0.50 | 7178 |
| Transferrin | YEYVTALQNLR | 1.91 | 0.46 | 9634 |
| Transferrin | YKELPDPQESIQ | 2.28 | 0.48 | 11522 |
| Transferrin | YKELPDPQESIQR | 2.19 | 0.46 | 19648 |
| Transferrin | YLGYEYVTALQNLR | 1.70 | 0.38 | 3158 |
| Transferrin | YVTALQNLR | 2.02 | 0.48 | 62340 |
| Transferrin | YYGYTGAFR | 1.19 | 0.33 | 277591 |
